# Linseed Cake Supplementation Increases Tissue n-3 PUFA Levels but Reduces Growth Performance in Broiler Chickens at Higher Inclusion Levels

**DOI:** 10.3390/life16010138

**Published:** 2026-01-15

**Authors:** Giedrius Šarauskas, Rasa Nainienė, Robertas Juodka, Artūras Šiukščius, Algirdas Urbšys, Monika Tiškutė, Raimondas Leikus

**Affiliations:** 1 Department of Animal Breeding and Reproduction, Animal Science Institute, Lithuanian University of Health Sciences, R. Zebenkos 12, 82317 Baisogala, Lithuania; arturas.siukscius@lsmu.lt (A.Š.); algirdas.urbsys@lsmu.lt (A.U.); 2 Department of Ecology, Animal Science Institute, Lithuanian University of Health Sciences, R. Zebenkos 12, 82317 Baisogala, Lithuania; robertas.juodka@lsmu.lt; 3 Analytical Laboratory, Animal Science Institute, Lithuanian University of Health Sciences, R. Zebenkos 12, 82317 Baisogala, Lithuania; monika.tiskute@lsmu.lt; 4 Department of Animal Feeding and Feedstuffs, Animal Science Institute, Lithuanian University of Health Sciences, R. Zebenkos 12, 82317 Baisogala, Lithuania; raimondas.leikus@lsmu.lt

**Keywords:** broiler chickens, linseed cake, fatty acid profile, n-3 polyunsaturated fatty acids, poultry meat, growth performance

## Abstract

This study evaluated the effects of dietary linseed cake on the fatty acid profile of meat and abdominal fat, and on growth performance in broiler chickens. A total of 198 birds were randomly allocated to three groups (66 birds/group). The control group (C) received a standard soybean meal-based feed, while the LIN6 and LIN12 groups were fed diets that were supplemented with 6% and 12% linseed cake, respectively. Linseed cake supplementation reduced saturated and monounsaturated fatty acids, increased n-3 polyunsaturated fatty acids (PUFAs) in meat and abdominal fat, and lowered the n-6/n-3 PUFA ratio (FDR-adjusted *p* < 0.05). The 12% inclusion resulted in a more pronounced accumulation of n-3 PUFAs—4.3–5.0 times higher than the control—while 6% inclusion increased n-3 PUFAs by 2.8–3.3 times (FDR-adjusted *p* < 0.05). However, 12% inclusion negatively affected growth performance, reducing body weight by 9.9% and feed intake by 10.4% at 42 days (*p* < 0.05), whereas the 6% inclusion had no adverse impact (*p* < 0.05). These results indicate that 6% linseed cake represents the optimal practical inclusion level, effectively enhancing the n-3 PUFA profile of broiler meat and abdominal fat without compromising growth, while higher inclusion levels may impair production performance.

## 1. Introduction

The decisions of modern consumers are shaped by nutritional value, health considerations, animal welfare, and environmental sustainability [1,2,3]. Diets rich in n-3 polyunsaturated fatty acids (PUFAs) are associated with reduced risks of cardiovascular, inflammatory, metabolic diseases, and depression [4,5,6]. However, typical Western diets remain extremely low in n-3 PUFAs, with n-6/n-3 ratios (15:1) far exceeding the recommended 1–2:1 [7].

Humans cannot synthesize α-linolenic acid (ALA; C18:3 n-3), which must therefore be obtained from dietary sources [8]. ALA also serves as the metabolic precursor for the long-chain n-3 PUFAs eicosapentaenoic acid (EPA; C20:5 n-3) and docosahexaenoic acid (DHA; C22:6 n-3), which are both essential to human health and development. However, this conversion is inefficient (<8% for EPA and <1% for DHA) [8,9]. Consequently, increasing the intake of ALA and long-chain n-3 PUFA-rich foods remains an important nutritional goal [10].

Studies indicate that poultry can efficiently convert dietary ALA into long-chain n-3 PUFAs, and the fatty acid composition of poultry diets directly affects the lipid profile of meat and eggs [11,12,13]. However, conventional poultry production systems typically involve feed formulations that are low in n-3 PUFAs [14,15,16,17,18,19], resulting in poultry products with low n-3 PUFA levels [11,12,13,20,21]. Consequently, because poultry meat is among the most widely consumed animal products globally, enriching it with n-3 PUFAs could improve population-level n-3 PUFA intake with little change in dietary habits.

Among plant-based sources of n-3 PUFAs, linseed is particularly promising due to its high ALA content, adaptability to low-input and organic farming systems, positive effects on soil structure and biodiversity, and lower environmental footprint [22,23,24,25,26,27]. In poultry nutrition, linseed oil, whole seeds, meal, and cake can be incorporated into feed formulations. Linseeds and their derived feed components have a similar fatty acid profile, with n-3 PUFAs predominating (>50%) and n-6 PUFAs around 9–20% [28,29,30,31,32,33,34,35].

Linseed oil and whole seeds are highly effective at increasing tissue n-3 PUFA levels. For example, supplementing broiler diets with 3–4% linseed oil increased ALA and total n-3 PUFA in meat up to eight- to nine-fold, with similar increases in abdominal fat [36,37]. Incorporating 10% whole linseed increased meat ALA six- to nine-fold, while in turkeys, total n-3 PUFAs increased by 1.7 times [25,36,37,38]. However, linseed oil and seeds are nutrient-dense foods for human consumption, and their use in feed raises sustainability and economic concerns [26].

These challenges have led to increasing attention on alternative linseed-based ingredients that can provide similar nutritional benefits while enhancing sustainability. In this context, linseed cake—a by-product of cold-pressed oil extraction—offers a promising yet less extensively studied option. Unlike oil and whole seeds, linseed cake is generally unsuitable for direct human consumption without further processing [39,40] but is a sustainable feed ingredient, with 28–35% crude protein, 5–29% residual oil, and ~51% ALA [25,39,40,41,42,43,44,45,46,47,48,49], with a favorable n-6/n-3 PUFA ratio of 0.30. Nevertheless, linseed cake retains anti-nutritional factors such as cyanogenic glycosides and mucilage, which may reduce nutrient digestibility at high inclusion levels [45,46,47,48,49], highlighting the need for further research to define optimal dietary inclusion rates.

Most previous studies have focused on linseed oil or meal. Inclusion of linseed meal at 10–26% has been shown to increase the total n-3 PUFAs by 1.37–2.53 times and reduce the muscle n-6/n-3 ratio to 2.05–2.60 [27,50,51]. However, the effects of linseed products on growth performance remain contradictory, with some studies reporting no adverse effects at 5–10% or even 15–20% inclusion [41,52,53,54,55,56], while others observe reduced weight gain at lower inclusion levels (4–5%) [57,58].

Linseed cake has clear potential to improve the fatty acid profile of poultry meat; however, very limited research has examined its effects on growth performance, nutrient utilization, or fatty acid composition in broilers [35,57]. This leaves a notable gap concerning optimal inclusion levels and the implications for meat quality. Addressing this gap is important from both nutritional and sustainability perspectives, as linseed cake provides a cost-effective means of enriching poultry meat with n-3 PUFAs while valorizing an agricultural by-product.

We hypothesized that increasing linseed cake inclusion in broiler diets would enhance the fatty acid profile of the meat and abdominal fat by increasing the n-3 PUFA content and lowering the n-6/n-3 ratio. We also expected that inclusion levels of 6% and 12% would have varying effects on growth performance.

Therefore, the objective of this study was to investigate the effect of including linseed cake in the chicken diet on the fatty acid profile changes in meat and abdominal fat, as well as on growth performance.

## 2. Materials and Methods

### 2.1. Poultry Management and Study Design

The investigations were conducted in accordance with Directive 2010/63/EU of the European Parliament and of the Council of 22 September 2010 on the protection of animals used for scientific purposes [59]. In addition, all procedures complied with the Law of the Republic of Lithuania on Animal Welfare and Protection (Law No. IX-2271) [60], and a sub-statutory act issued by the State Food and Veterinary Service of the Republic of Lithuania concerning the approval of requirements for the use, research, storage, maintenance, and handling of experimental animals [61]. The experiment was approved by the Board of the Animal Science Institute of the Lithuanian University of Health Sciences (Protocol No. 24/01/30/01, dated 30 January 2024).

A total of 198 chickens of Ross 308 cross were obtained from a commercial hatchery as hatched. The birds were not sexed and were randomly assigned to three dietary treatment groups (66 birds each) with six replicates of 11 chickens each. The trials with chickens began at 15 days of age (initial weight: 582.09 ± 4.89 g) and concluded at 42 days of age (final weight: 2907.56 ± 32.01 g), spanning a total duration of 28 days. Up to 14 days of age, all chickens were fed a standard crumbled starter compound feed. From day 15 onward, chickens in the control group (C) received a pelleted compound feed with soybean meal as the main protein source (Table 1). The treatment groups, LIN6 and LIN12, were fed pelleted diets supplemented with 6% and 12% linseed cake, respectively. The compound feeds for all groups were isoprotein and met the energy and nutrient requirements of broiler chickens, according to the Ross Broiler: Nutrition Specifications [62]. The nutritive values of linseed cake are given in Table 2. Linseed cake obtained from linseed oil extraction by cold mechanical pressing—a solvent-free method—was used in the experiment. The oil was extracted using a KEK-P0020 cold press (KEK EGON KELLER, Remscheid, Germany) with a processing capacity of 30 kg/h, operating at 20–25 °C. The press is a single-screw design equipped with a 2.2 kW motor.

During the experimental period, water and feed were provided ad libitum. All the birds were reared under the same conditions. Chickens were housed in enclosures measuring 3.24 m^2^ (1.80 × 1.80 m), with 11 birds per enclosure, providing 0.295 m^2^ per bird. The temperature mode for chicken rearing under the brooder was as follows: 32 °C, 30 °C, 28 °C, 27 °C, and 26 °C for the chickens of, respectively, one, three, six, nine, and twelve days of age. When the chickens reached the age of two weeks, the temperature in the poultry house was 24 °C, at the age of three weeks it was 22 °C, and from the age of four weeks until the end, the temperature was 20 °C.

The relative humidity in the chicken room was 60–65%. The light–dark schedules were 23L:1D. Light intensity was varied throughout the experimental phases, with levels set at 35 lux for days 0–7, 20 lux for days 8–21, and 10 lux for days 21–42, utilizing white light.

The birds were weighed individually at 14, 21, 28, 35, and 42 days of age. Feed ac-counting was carried out by individually weighing the feed for each replicate before feeding. Electronic scales KB-6000 with a weighing error of 1g and at maximum weighing up to 15 kg were used to weigh chickens and feeds.

Feed conversion ratio (FCR) was calculated by dividing the total feed consumed (kg) during the analyzed period by the total weight gain (kg), according to the following formula:

FCR = Total Feed Consumed (kg)/Total Weight Gain (kg).

### 2.2. Carcass and Meat Quality Evaluation

At the end of the study (at age of 42 days), 24 chickens (12 males and 12 females) from each group were selected for control slaughtering. Prior to slaughter, the birds had not been fed for 12 h and afterwards were slaughtered in a commercial EU-licensed abattoir. Carcasses were anatomically dissected according to the methodological recommendation [63]. At slaughter, breast and leg muscle samples (200 g) were taken from each bird for the chemical composition analysis. Dry matter, protein, fat, and ash were analyzed in the breast and leg muscle according to AOAC methods [64].

### 2.3. Chemical Analyses

#### 2.3.1. Chemical Analyses of the Feed and Tissues

The feeds were analyzed for the dry matter content by oven drying at 105 °C up to a constant weight; the crude protein content was determined by the Kjeldhal method; crude fat was analyzed by the extraction method using petroleum ether; crude fiber was analyzed by the Fibercap method; crude ash was determined by the gravimetric method by burning samples in a muffle furnace at a temperature of 500–550 °C [64]; calcium was analyzed by the atomic absorption method, using cesium chloride and aluminum nitrate; phosphorus was analyzed by the photometric method, using a molybdovanadate reagent [64]; nitrogen-free extractive matter (NEM) was analyzed by the difference after deducting the amounts of crude protein, fat, fiber, and ash from the dry matter expressed in %; amino acids were analyzed by the efficient liquid chromatography method (AccQ Tag method; Waters Corp., Milford, MA, USA). Metabolizable energy in MJ/kg was calculated according to the chemical analysis data of the feed and the digestibility coefficients indicated in the literature [65]. The tissues’ crude fat was analyzed by the Soxhlet extraction method.

#### 2.3.2. Fatty Acid Analyses

The content of fatty acids was analyzed in feeds given to chickens in both rearing periods. At slaughter, 50 g of breast muscle (*musculus pectoralis major*) and thigh muscles (*musculus iliotibialis lateralis postacetabularis and musculus flexor cruris lateralis pars pelvica*), as well as 10 g of abdominal fat, were collected from each chicken for fatty acid composition analysis.

Tissue samples were collected and homogenized using an IKA T18 Digital UL-TRA-TURRAX^®^ homogenizer (IKA, Staufen, Germany) with digital speed control (3000–25,000 rpm), ensuring uniform sample preparation.

Lipids were extracted for fatty acid analysis according to the method described by Folch et al. [66], using a 2:1 (*v*/*v*) mixture of chloroform (Chromasolv Plus for HPLC with 0.5–1.0% ethanol) and methanol (Chromasolv for HPLC, ≥99.9%). The method described by Christopherson and Glass [67] was used to prepare fatty acid methyl esters (FAME) from the total lipids. A Shimadzu GC-2010 Plus gas chromatograph (Shimadzu Corp., Kyoto, Japan) fitted with a flame ionization detector was used to analyze the FAME. A capillary column Rt-2560 (100 m; 0.25 mm ID; 0.25 µm df) from Restek (Bellefonte, PA, USA) was used to separate FAME, using temperature programming ranging from 160 °C to 230 °C. The detector and injector temperatures were kept at +260 °C and +240 °C, respectively. The flow rate of nitrogen, the carrier gas, was fixed at 0.79 mL/min. The injection volume was 1.0 µL, and the total gas chromatography run time was 60 min. Fatty acid methyl esters (FAMEs) were identified by comparing their retention times with those of authentic standard mixtures (Supelco 37 Component FAME Mix, Merck KGaA, Darmstadt, Germany). The relative proportion of each fatty acid in the sample was expressed as a percentage of the total fatty acid content, using LAB Solutions LC/GC software (version 5.71) for the Shimadzu GC-2010 Plus gas chromatograph.

The following lipid quality indices were calculated based on fatty acid composition data: the atherogenic index (AI) and thrombogenic index (TI) [68], the hypocholesterolemic/hypercholesterolemic index (h/H) [69], the peroxidability index (PI) and unsaturation index (UI) [70], the desirable fatty acids (DFA) index [71], and the hypercholesterolemic saturated fatty acids (HSFA) index [72].

AI = (C12:0 + 4 × C14:0 + C16:0)/(PUFA + MUFA);

TI = (C14:0 + C16:0 + C18:0)/(0.5 × MUFA + 0.5 × n-6 PUFA + 3 × n-3 PUFA + n-3/n-6 PUFA);

h/H = (C18:1n-9 + C18:2n-6 + C18:3n-3 + C20:3n-3 + C20:4n-6 + C20:5n-3 + C22:4n-6 + C22:5n-3 + C22:6n-3)/(C14:0 + C16:0);

PI = (% monoenoic × 0.025) + (% dienoic × 1) + (% trienoic × 2) + (% tetraenoic × 4) + (% pentaenoic × 6) + (% hexaenoic × 8);

UI = 1 × (% monoenoics) + 2 × (% dienoics) + 3 × (% trienoics) + 4 × (% tetraenoics) + 5 × (% pentaenoics) + 6 × (% hexaenoics);

DFA = UFA + C18:0;

UFA = MUFA + PUFA;

HSFA = C12:0 + 4 × C14:0 + C16:0.

#### 2.3.3. Chemicals Used for Analyses

Crude protein content was determined by using sulfuric acid, hydrochloric acid, boric acid, sodium hydroxide, and Kjeltabs ST. Crude fat content was determined by using petroleum ether (b.p. 40–60 °C) and anhydrous sodium sulfate. Fatty acid identification was performed using the 37 Component FAME Mix and trans FAME Mix K 110 standards (Merck KGaA, Darmstadt, Germany).

All reagents, except Kjeltabs ST (VELP Scientifica, Via Stazione, Usmate Velate, Italy) and fatty acid standards (Supelco Analytical, Bellefonte, PA, USA), were purchased from Sigma-Aldrich Co. (St. Louis, MO, USA).

### 2.4. Statistical Analyses

A priori power analysis was performed in IBM SPSS Statistics (version 30) for a one-way ANOVA with three groups. Assuming a medium effect size (f = 0.25), α = 0.05, and power = 0.80, the required sample size was 158 individuals (≈53 per group). To account for potential bird losses and to maintain even cage stocking density, 66 birds per group were included in the experiment.

Data normality was tested by using the Shapiro–Wilk test and visual inspection of histograms and boxplots. Normally distributed variables (growth performance, carcass traits, and meat composition) were analyzed by the general linear model (GLM, full factorial) in SPSS with Tukey’s post hoc test. Results are expressed as mean ± standard error of the difference (SED).

Most fatty acid data were not normally distributed and were analyzed using the Kruskal–Wallis test, followed by Dunn’s pairwise comparisons. To control for multiple testing, the *p*-values were adjusted by the Benjamini–Hochberg false discovery rate (FDR) method. Although non-parametric tests were applied, fatty acid data are presented as mean ± SE for consistency. Values within a row bearing different superscript letters differ significantly (FDR-adjusted *p* < 0.05).

## 3. Results and Discussion

### 3.1. Growth Performance

The inclusion of 6% linseed cake (LIN6) in broiler chicken diets did not significantly impact growth performance compared to the control group (Table 3). In contrast, the 12% inclusion level (LIN12) resulted in a marked reduction in live body weight and feed intake, with body weight at 42 days being 9.9% lower and feed intake 10.4% lower than in the control group (*p* < 0.05). This indicates that high levels of linseed cake can negatively affect growth, which is likely due to reduced palatability and the presence of anti-nutritional compounds [41,73,74]. Although the feed conversion ratio (FCR) remained unaffected, the decline in feed intake and body weight at the higher inclusion level represents a critical limitation for practical application.

The negative effects of linseed cake can be attributed to its content of various antinutritional factors, including the cyanogenic glycosides, linatine, trypsin inhibitors, phytic acid, mucilage, and high levels of non-starch polysaccharides (NSPs) [25,41,47,49]. As the inclusion level of linseed cake increases, the concentrations of these compounds rise correspondingly, eventually reaching levels that can impair growth performance.

Phytic acid decreases mineral bioavailability, impairs protein absorption, and inhibits proteolytic enzyme activity, while NSPs increase the jejunal digesta viscosity, thereby reducing nutrient absorption efficiency [25,41,47,49,55,75]. Research has shown that poultry that are fed linseed-rich diets exhibit lower digestibility of organic matter (OM), dry matter (DM), crude protein (CP), and crude fat (CF), which in turn contributes to reduced growth performance [45,55,76,77]. These findings underscore the practical implications for nutrient-use efficiency when higher levels of linseed cake are incorporated into poultry diets.

These results are consistent with previous studies reporting growth suppression at elevated levels of linseed or linseed-derived products in poultry diets [25,73,75,78]. In contrast, and as demonstrated in several other studies, supplementation of the birds’ diet with linseed oil has generally not been associated with negative effects on body weight [79,80,81], suggesting that the adverse effects observed here are specific to the non-lipid components of linseed cake.

From a practical perspective, the present findings indicate that linseed cake can be effectively included at moderate levels (up to 6%) to partially replace soybean meal without adverse effects on performance. However, higher inclusion levels (≥12%) are not advisable under standard production conditions, due to their negative impact on growth. While no mitigation strategies were applied in this study, future research could explore enzyme supplementation, heat treatment, or other feed-processing methods to reduce anti-nutritional effects and improve nutrient availability in linseed-containing broiler diets.

Overall, the ability to include linseed cake at moderate levels offers an opportunity to improve feed sustainability and reduce dependence on conventional protein sources, provided that inclusion rates are carefully controlled.

### 3.2. Carcass Dissection Data and Meat Chemical Composition

Linseed cake inclusion also affected carcass characteristics. The dressing percentage decreased by 1.24% in the LIN6 group and by 1.7% in the LIN12 group (*p* < 0.05; Table 4). Moreover, LIN12 carcasses were 11.61% lighter than those in the control group. The proportion of non-edible internal parts increased by 0.48% in the LIN6 group and by 1.06% in the LIN12 group (*p* < 0.05).

Despite these changes, the proportions of valuable muscle groups (breast, thigh, drumstick) and internal organs (heart, liver, gizzard, and abdominal fat) remained unaffected by dietary treatment.

Chemical composition of breast and leg muscles showed minimal variation (Table 5). Dry matter, protein, and ash content in breast muscles did not differ significantly across groups, although the fat content was slightly lower (*p* < 0.05) in the LIN6 and LIN12 groups (by 0.27–0.47%). Similarly, leg muscles in the LIN12 group did not show significant differences, while a small reduction in ash content (0.08%) was noted in the LIN6 group (*p* < 0.05).

### 3.3. Fatty Acid Profiles of Intramuscular Fat in the Breast and Thigh Muscles and Abdominal Fat of Broiler Chicken

#### 3.3.1. Saturated Fatty Acids

The inclusion of linseed cake in the diet of broiler chickens reduced the total saturated fatty acid (SFA) content of the feed (Table 2), which was reflected in significantly lower total SFA levels in the tissues of chickens in the LIN6 and LIN12 groups, compared with the control (FDR-adjusted *p* < 0.05; Table 6, Table 7 and Table 8).

The reduction in total SFA content is nutritionally relevant, as a high dietary SFA intake increases LDL cholesterol and the risk of cardiovascular disease [82], as well as thrombosis, inflammation, and oxidative stress [83]. This change was primarily attributed to a significant decrease in palmitic acid (C16:0) (FDR-adjusted *p* < 0.05), the predominant fatty acid in the SFA group (Table 6, Table 7 and Table 8), which has been associated with coronary heart disease [84]. Specifically, palmitic acid (C16:0) levels (FDR-adjusted *p* < 0.05) were 1.12–1.25 times lower in the meat and abdominal fat of birds in the LIN6 and LIN12 groups.

A consistent decline in myristic acid (C14:0) (FDR-adjusted *p* < 0.05)—a highly atherogenic SFA [85,86,87]—was also observed across all tissues, which was likely due to its lower concentration in the finishing diets.

Additional reductions were observed for behenic acid (C22:0) in muscle tissues (FDR-adjusted *p* < 0.05) and margaric acid (C17:0) (FDR-adjusted *p* = 0.040) in abdominal fat.

Despite the general downward trend in total SFA levels, certain individual SFAs—lauric (C12:0) (FDR-adjusted *p* = 0.039), pentadecanoic (C15:0) (FDR-adjusted *p* = 0.039), stearic (C18:0) (FDR-adjusted *p* = 0.039), and arachidic (C20:0) (FDR-adjusted *p* = 0.039) acids—showed increased levels in the breast muscle, indicating selective fatty acid deposition and metabolism in different tissues.

However, although the dietary intake of arachidic fatty acid (C20:0) was lower, its accumulation in the breast muscle was higher (FDR-adjusted *p* = 0.039), which is relevant given its association with a reduced risk of coronary heart disease [88] and type 2 diabetes mellitus [89].

These findings are consistent with previous studies, such as Tamasgen et al. [51], who reported similar reductions in total SFAs, particularly palmitic acid, following dietary supplementation with 6.5–26% linseed meal.

The current results suggest that incorporating linseed cake into broiler diets reduces harmful SFAs, such as palmitic (C16:0) and myristic acids (C14:0), and enhances beneficial components like arachidic acid (C20:0).

#### 3.3.2. Monounsaturated Fatty Acids

In the thigh muscles and abdominal fat of birds from the LIN6 and LIN12 dietary groups, levels of oleic acid (C18:1n-9) were significantly lower (FDR-adjusted *p* = 0.040) than in the control group, while palmitoleic acid (C16:1n-7) levels significantly decreased (FDR-adjusted *p* < 0.05) across all examined tissues (Table 6, Table 7 and Table 8). This reduction contributed to a 1.07- to 1.26-fold (FDR-adjusted *p* < 0.05) decrease in total monounsaturated fatty acid (MUFA) content (Table 6, Table 7 and Table 8).

The decline in MUFAs—particularly oleic acid—may be considered nutritionally unfavorable, given their established role in supporting healthy lipid metabolism and cardiometabolic function [90,91]. These findings align with previous studies reporting similar reductions in MUFAs in poultry fed diets enriched with n-3 PUFAs, such as linseed or linseed products. The decreased MUFA levels are likely a result of inhibited Δ9-desaturase activity due to the elevated dietary intake of n-3 PUFAs, which suppress de novo MUFA synthesis in body tissues [92]. Comparable reductions in oleic acid (C18:1n-9) have been documented in chicken tissues with 26% linseed meal [51], those with 3–4% linseed oil [37,93], and in turkeys with 2.5% linseed oil [25].

However, several studies did not observe these effects. Chen et al. [52] reported no significant changes in oleic acid or total MUFA levels in chicken breast muscle with 5% linseed oil, and Jankowski et al. [25] similarly found no effect on breast muscle MUFA levels in turkeys supplemented with 2.5% linseed oil. Taken together, these findings suggest that alterations in MUFA composition are less likely when linseed is provided in oil form at moderate inclusion levels, whereas more pronounced effects may occur with higher inclusion rates or when linseed is supplied in less refined products (e.g., cake or meal) that contain non-lipid components and are fed for longer periods.

The response of individual MUFAs showed tissue specificity. Eicosenoic acid (C20:1n-9) content in the breast muscles of the LIN6 and LIN12 birds increased significantly (FDR-adjusted *p* = 0.039), reaching levels 2.3 to 2.6 times higher than in controls (Table 6). Conversely, in the thigh muscles, eicosenoic acid (C20:1n-9) levels were 1.3 to 1.4 times lower (FDR-adjusted *p* = 0.040) than in the control group, indicating a tissue-specific redistribution or metabolic modulation of MUFAs under the influence of linseed cake supplementation (Table 7).

The analysis detected low concentrations of erucic acid (C22:1n-9) in the breast (0.04–0.06%), thigh (0.01–0.02%), and abdominal fat (0.02%). MUFA is generally considered undesirable due to its known cardiotoxic effects at a high concentration [94]. Compared with the control group, birds that were fed the LIN6 and LIN12 diets exhibited significantly higher levels of erucic acid (C22:1n-9) (FDR-adjusted *p* < 0.05). Although the LIN12 group showed up to a sixfold increase in erucic acid (C22:1n-9) content in breast muscle compared to the controls (FDR-adjusted *p* = 0.039), the absolute concentrations across all tissues remained very low and far below the European Union’s maximum limit of 5% in food products [95]. In addition, the thigh muscle of the LIN12 group had erucic acid (C22:1n-9) levels that were three times lower than in the control group (FDR-adjusted *p* = 0.040). These results suggest that while linseed cake does introduce erucic acid (C22:1n-9) into the diet, the levels observed are minimal and pose no health risks, according to the current regulatory thresholds [94].

The reduction in MUFA, combined with the minor presence of erucic acid, highlights potential trade-offs in meat fatty acid composition and should be considered when evaluating the nutritional quality of broiler meat.

#### 3.3.3. Polyunsaturated Fatty Acids

n-6 PUFA. The inclusion of linseed cake altered the n-6 PUFA profile and its deposition in chicken tissues. Although dietary n-6 PUFA intake decreased by 1.2-fold in LIN6 and 1.4-fold in LIN12 compared with the control (Table 1), the total n-6 PUFA deposition increased slightly (1.05–1.08-fold; FDR-adjusted *p* < 0.05) in both experimental groups (Table 9, Table 10 and Table 11).

Breast and abdominal fat showed increased levels of linoleic acid (LA; C18:2n-6) (FDR-adjusted *p* < 0.05), while γ-linolenic acid (C18:3n-6) was reduced in the breast and thigh muscles (Table 9, Table 10 and Table 11) (FDR-adjusted *p* < 0.05). Additionally, breast muscle of birds from the exhibited experimental groups contained higher arachidonic (C20:4n-6) (FDR-adjusted *p* = 0.039) acid (Table 9). (*p* = 0.039) acid (Table 9).

However, increasing the linseed cake from 6% to 12% did not further alter the n-6 PUFA profile, and LIN6 and LIN12 did not differ significantly in the total n-6 PUFA, LA, or γ-linolenic (C18:3n-6) acids (Table 9, Table 10 and Table 11).

These results indicate a metabolic adaptation in chickens whereby linseed cake, de-spite its high ALA content, indirectly enhances the tissue accumulation of certain n-6 PUFAs. This is consistent with Nain et al. [96], who reported that higher dietary ALA stimulates both LC n-3 PUFA synthesis and endogenous n-6 PUFA production. Jankowski et al. [25] similarly observed increased n-6 PUFA levels in turkey meat following dietary supplementation with 2.5% linseed oil.

Contrarily, other studies have reported reductions or no changes in total n-6 PUFA following linseed supplementation. Kanakri et al. [37] and Tamasgen et al. [51] observed decreased n-6 PUFA levels with linseed oil and linseed meal, respectively, whereas Nguyen et al. [36] reported no significant effects. These discrepancies may be attributed to differences in study design, linseed form, and supplementation level.

n-3 PUFA. The inclusion of linseed cake in broiler diets promoted the accumulation of n-3 PUFAs in chicken tissues, particularly with the most notable increase observed in ALA (Table 9, Table 10 and Table 11). ALA levels in the breast, thigh muscles, and abdominal fat increased by 3.2-, 3.4-, and 3.7-fold (FDR-adjusted *p* < 0.05) in the LIN6 group and by 5.0-, 5.4-, and 5.8-fold (FDR-adjusted *p* < 0.05) in the LIN12 group, compared to the control, with the LIN12 group exhibiting approximately 1.6 times higher (FDR-adjusted *p* < 0.05) ALA levels than the LIN6 group (Table 9, Table 10 and Table 11).

ALA, an essential n-3 PUFA, must be supplied through the diet, as chickens cannot synthesize it. In this study, supplementation with 6% and 12% linseed cake increased the ALA content in the feed by 2.9–3.3-fold and 4.7–5.0-fold, respectively. This led to the accumulation not only of ALA but also of LC n-3 PUFAs in the tissues (FDR-adjusted *p* < 0.05), as ALA serves as a precursor for LC n-3 PUFAs. Higher dietary ALA promote its bioconversion to LC n-3 PUFAs through desaturation and elongation processes [96,97,98,99,100].

As a result of these metabolic shifts, the accumulation of LC n-3 PUFAs—eicosatrienoic acid (ETE, C20:3n-3), docosapentaenoic acid (DPA, C22:5n-3), and docosahexaenoic acid (DHA, C22:6n-3)—increased markedly in chicken muscles (Table 9, Table 10 and Table 11). In the LIN6 group, increases ranged from 1.7- to 3.3-fold (FDR-adjusted *p* < 0.05), and in LIN12 from 2.1- to 4.6-fold (FDR-adjusted *p* < 0.05) in muscles. Furthermore, when comparing the two supplemented groups, the LIN12 group exhibited 1.5–1.6 times higher levels of DHA and DPA (FDR-adjusted *p* < 0.05) in breast muscle than the LIN6 group, indicating a dose-dependent effect of linseed cake supplementation on n-3 PUFA deposition.

Abdominal fat showed no significant accumulation of ETE and DHA, but EPA and DPA levels increased by 1.5–3.3 times (FDR-adjusted *p* = 0.040), indicating a lower deposition of LC n-3 PUFAs in fat compared to muscle tissue (Table 11).

The total LC n-3 PUFA increased 2.6–3.8-fold in breast, 1.9–2.2-fold in thigh, and 1.4–2.1-fold in abdominal fat (FDR-adjusted *p* < 0.05; Table 9, Table 10 and Table 11). LIN12 birds showed 1.2–1.5-fold higher total and LC n-3 PUFA than LIN6 (FDR-adjusted *p* < 0.05). Similar increases have been reported by Kanakri et al. [93] with 3% linseed oil.

The total n-3 PUFA deposition increased significantly in muscles—3.0–4.6-fold in breast and 3.1–4.7-fold in thigh (FDR-adjusted *p* < 0.05; Table 9, Table 10 and Table 11)—in the present study. A strong relationship between dietary and tissue n-3 PUFA content has also been reported by Kanakri et al. [37], who observed high Pearson correlation coefficients (r = 0.998–1.000). Similar increases in total n-3 PUFA levels in poultry muscle and abdominal fat following linseed or linseed oil supplementation have been reported by Chen et al. [52], Jankowski et al. [25], Kanakri et al. [93], and Nguyen et al. [36].

Our study confirms that a higher dietary inclusion of linseed cake led to more pronounced changes in the n-3 PUFA profile. This supports previous evidence that a lower dietary LA/ALA ratio upregulates hepatic and muscle gene expression related to lipid metabolism (FADS1, FADS2, ELOVL2, ELOVL5) [79,101]. In our study, supplementation with 6% linseed cake reduced the dietary LA/ALA ratio to 3.36–3.04, while 12% linseed cake further decreased it to 1.72 (Table 2). This shift contributed to a greater accumulation of n-3 PUFAs in broiler tissues. Correspondingly, the LA/ALA ratio in the breast and thigh tissues decreased to 4.89 and 4.69 in LIN6, and to 3.18 and 2.98 in LIN12, while in the control group, it was 3 to 5.1 times higher (FDR-adjusted *p* < 0.05; Table 9, Table 10 and Table 11).

In our study, the treated groups showed a reduction in total trans fatty acid content in their tissues. Trans fatty acid levels decreased most markedly in the LIN12 group, with a 1.4-fold reduction in thigh muscle and abdominal fat (FDR-adjusted *p* = 0.04; Table 10 and Table 11). Given the well-documented health risks associated with trans fatty acids—including elevated LDL cholesterol, reduced HDL cholesterol, and increased risk of type 2 diabetes [102]—this reduction may be considered a valuable nutritional benefit of dietary linseed cake supplementation.

The inclusion of linseed cake markedly reduced the n-6/n-3 PUFA ratio in chicken diets, reaching 3.04–3.37 in the LIN6 and 1.75–1.76 in the LIN12 feeds (Table 1), with corresponding changes in the tissue fatty acid composition. The most pronounced reduction was observed in the abdominal fat of LIN12 chickens, where the n-6/n-3 PUFA ratio decreased fivefold to 2.81 (FDR-adjusted *p* = 0.040; Table 11), while the smallest reduction occurred in the breast muscle of LIN6 birds (2.8-fold; FDR-adjusted *p* = 0.039; Table 9).

In LIN12 chicken meat, the n-6/n-3 PUFA ratio declined by 4.3–4.5 times (FDR-adjusted *p* < 0.05), reaching 2.85–2.94, which falls within the recommended range for a healthy diet [103]. These results agree with previous studies reporting similar improvements in poultry tissues following linseed supplementation [25,36,37,93,99].

#### 3.3.4. Indices

The atherogenic index (AI) decreased across all analyzed tissues of chickens that were fed linseed cake (FDR-adjusted *p* < 0.05; Table 12). According to Fernandes et al. [104], an AI value below 1 is indicative of favorable lipid profiles and is associated with a reduced risk of coronary artery disease. In our study, the LIN12 group showed the most pronounced improvement, with AI values ranging from 0.25 to 0.28 (FDR-adjusted *p* < 0.05). These results suggest a substantial enhancement in the nutritional quality of the lipid fraction.

A reduction in AI was also observed in the LIN6 group compared with the control, with values decreasing to 0.30–0.27 (FDR-adjusted *p* < 0.05). Notably, the AI values obtained in both linseed-fed groups were lower than those reported by Kumar et al. [27], who observed AI values between 0.33 and 0.67 in chickens receiving diets containing 10% linseed meal. This indicates that the inclusion of linseed cake, particularly at higher levels, may have a more favorable effect on decreasing the atherogenic potential of chicken tissues than the linseed meal reported in previous studies.

The thrombogenic index (TI) also declined across all tissues, and all TI values fell below 0.5, a range considered desirable in health-promoting food [104]. In the present study, the most favorable TI values were recorded in the LIN12 muscle tissues (0.37–0.43) and in the abdominal fat (0.31) (FDR-adjusted *p* < 0.05; Table 12). Comparable improvements were reported by Kumar et al. [27], who observed a decrease in TI from 1.04 to 1.20 to 0.41–1.09 following dietary inclusion of linseed meal.

The hypocholesterolemic/hypercholesterolemic fatty acid ratio (h/H index), which reflects the balance of beneficial to harmful fatty acids, increased in all tissues of chickens that were fed LIN6 and LIN12 diets (FDR-adjusted *p* < 0.05; Table 12). A value above two is considered optimal [105]; the h/H index observed in our study demonstrates clear improvements in the lipid quality. Specifically, the h/H index in the LIN6 group ranged from 3.17 to 3.50, while values in the LIN12 group ranged from 3.40 to 3.95 (FDR-adjusted *p* < 0.05). These findings are consistent with previous studies by Gheorghe et al. [106] and Kralik et al. [107], who also reported increases in the h/H index following the inclusion of extruded linseed or linseed oil in poultry diets.

The desirable fatty acids (DFA) index also increased in all LIN6 and LIN12 tissues, with the greatest improvements noted in the LIN12 thigh and abdominal fat (1.06-fold) (FDR-adjusted *p* < 0.05; Table 12). We observed the highest overall DFA index in the LIN12 abdominal fat (81.89) (FDR-adjusted *p* = 0.040; Table 12).

Furthermore, the index of hypercholesterolemic saturated fatty acids (HSFA) decreased with increasing linseed cake inclusion. The most significant reductions were observed in the LIN12 abdominal fat (1.24-fold) and thigh muscle (1.23-fold), with the lowest HSFA values recorded in the LIN12 abdominal fat (18.50) and thigh muscle (18.59) (FDR-adjusted *p* < 0.05; Table 12), further supporting the positive impact of linseed cake on the lipid quality.

Dietary linseed supplementation caused a clear, dose-dependent increase in the peroxidability index (PI) of all examined chicken tissues (Table 12). Breast muscle PI increased from 42.07 in the control group to 57.74 in LIN6 and 70.05 in LIN12 (FDR-adjusted *p* = 0.039). Thigh muscle showed a similar pattern (40.18, 51.87, and 58.69; FDR-adjusted *p* = 0.040). Abdominal fat displayed the lowest absolute PI values but the same linear response (32.08, 43.11, and 50.82; FDR adj. *p* = 0.040).

These results indicate that linseed cake effectively increases tissue PUFA deposition, particularly n-3 fatty acids, which elevates its susceptibility to lipid peroxidation [108]. The magnitude of this effect was tissue-dependent. Breast muscle showed the highest PI values, reflecting its greater phospholipid content, which incorporate long-chain PUFA efficiently [109]. Thigh muscle exhibited moderate PI levels, while abdominal fat showed the lowest PI, which is consistent with its triacylglycerol-rich composition, which is dominated by shorter-chain fatty acids and contains fewer highly unsaturated lipid species [110].

Similar PI values have been reported in other studies [111,112]. Muscle PI in broilers ranges from 57.3 to 72.18 in conventional diets [112,113] and varies with each rearing system: 50.89–59.87 for indoor, 54.52–67.11 for outdoor, and 53.50–59.15 for organic production [111,114].

Overall, the results confirm that increasing levels of dietary linseed markedly increase the peroxidability of broiler tissues, with the strongest effects observed in lean, phospholipid-rich muscles. However, as shown in many studies, higher PI values are also associated with greater protective potential against coronary artery disease [115]. Therefore, it may be assumed that the higher PI values observed in the LIN6 and LIN12 groups may indicate an enhanced pro-health value for the tissues obtained from these birds. Consequently, to reduce the susceptibility of these PUFA-enriched tissues to peroxidation, special oxidation-control technologies are generally required when the peroxidability index exceeds approximately 60–65, and they become essential above approximately 70, particularly for n-3 enriched poultry meat. These antioxidant strategies include increased dietary vitamin E supplementation or the use of natural plant extracts, as well as specialized packaging technologies such as vacuum packaging or modified-atmosphere packaging with increased CO_2_ and reduced O_2_.

## 4. Conclusions

The inclusion of linseed cake—a by-product of cold oil extraction—in broiler chickens’ diets significantly improved the fatty acid composition profile of breast meat, thigh meat, and abdominal fat. Partial replacement of soybean meal with linseed cake notably increased the levels of health-promoting n-3 PUFAs, particularly α-linolenic acid and long-chain n-3 PUFAs, while simultaneously reducing the n-6/n-3 PUFA ratio, saturated and monounsaturated fatty acids, and trans fatty acids (FDR-adjusted *p* < 0.05). Consequently, key nutritional indices improved, including lower AI, TI, and HSFA values and a higher h/H ratio and PI.

Although higher dietary inclusion enhanced n-3 PUFA deposition, this benefit was accompanied by notable drawbacks. The 12% inclusion level produced the greatest increase in tissue n-3 PUFAs (4.3–5.0-fold higher than the control; FDR-adjusted *p* < 0.05), but it also significantly impaired the growth performance (*p* < 0.05), indicating that excessive inclusion compromises production outcomes. In contrast, the 6% inclusion level maintained normal growth and feed utilization while still substantially improving tissue n-3 PUFA deposition (2.8–3.3-fold above the control; FDR-adjusted *p* < 0.05) demonstrating a more appropriate balance between nutritional enhancement and productive performance.

Overall, these findings demonstrate that 6% linseed cake represents the optimal practical inclusion level, effectively balancing significant enrichment of poultry meat with n-3 PUFAs and the maintenance of the growth performance. At this level, linseed cake can serve as a sustainable and effective alternative source of dietary protein and fat in chicken nutrition, improving the health value of poultry meat without compromising productivity. Broader adoption of linseed cake could reduce reliance on imported, less sustainable soybean meal and strengthen local linseed production, depending on regional linseed availability, production capacity, and economic feasibility, thereby contributing to agricultural sustainability and crop diversification.

Nevertheless, the current findings highlight the need for further research to address the performance-limiting effects of higher inclusion levels. Future studies should also explore natural dietary antioxidants or other strategies that are capable of reducing the peroxidability index of PUFA-enriched poultry meat.

## Figures and Tables

**Table 1:** Diet composition used in the trial.

Specification	15–28 Days of Age (Grower)			29–42 Days of Age (Finisher)		
	Group					
	C	LIN6	LIN12	C	LIN6	LIN12
Wheat, %	41.82	39.50	37.15	47.52	45.20	42.88
Maize, %	20.00	20.00	20.00	20.00	20.00	20.00
Soybean meal, %	28.05	24.40	20.76	19.83	16.18	12.53
Sunflower meal, %	5.00	5.00	5.00	8.00	8.00	8.00
Linseed cake, %	0.00	6.00	12.00	0.00	6.00	12.00
Premix, % *	0.50	0.50	0.50	0.50	0.50	0.50
Feeder’s chalk, %	1.47	1.51	1.55	1.21	1.25	1.29
Monocalcium phosphate, %	0.49	0.41	0.34	0.32	0.24	0.16
Vegetable oil, %	2.00	2.00	2.00	2.00	2.00	2.00
L-lysine %	0.13	0.17	0.21	0.16	0.20	0.24
DL-methionine, %	0.44	0.41	0.39	0.36	0.33	0.30
Fodder salt, %	0.10	0.10	0.10	0.10	0.10	0.10
Calculated nutritional value of feed mixture (in kg)						
Dry matter, kg	0.871	0.873	0.876	0.869	0.871	0.874
Metabolizable energy, MJ	12.29	12.42	12.54	12.34	12.47	12.59
Crude protein, g	215.03	215.03	215.03	195.07	195.07	195.06
Lysine (dig.), g	11.61	11.58	11.55	10.31	10.28	10.25
Methionine + Cystine (dig.), g	9.24	9.20	9.22	8.35	8.33	8.29
Threonine (dig.), g	7.55	7.59	7.63	6.84	6.87	6.91
Tryptophan (dig.), g	1.97	2.06	2.14	1.84	1.92	2.01
Analyzed nutritional value of feed mixture (in kg)						
Dry matter, kg	0.884	0.877	0.888	0.883	0.881	0.885
Crude protein, g	216.66	215.94	216.12	196.42	195.85	195.78
Fat, g	35.54	44.43	56.11	36.11	45.64	55.51
Fiber, g	39.52	46.30	55.21	38.93	47.45	58.35
Ash, g	42.33	42.27	43.80	37.11	35.67	35.24
Calcium, g	9.52	9.41	9.49	8.18	8.09	8.14
Phosphorus, g	5.47	5.33	5.40	5.16	5.05	5.09
Fatty acids (% of total fatty acids)						
Total SFA	15.06	14.04	13.19	14.64	13.64	13.05
Total MUFA	22.43	22.03	21.66	23.03	21.64	21.11
n-6 PUFA	57.34	49.29	41.52	57.33	48.70	41.91
Linoleic (C18:2n-6)	56.79	48.78	41.03	56.82	47.98	41.16
n-3 PUFA	5.17	14.64	23.63	5.00	16.02	23.93
α-linolenic (C18:3n-3)	4.98	14.45	23.46	4.74	15.77	23.68
Eicosapentaenoic (C20:5n-3)	0.16	0.15	0.13	0.16	0.12	0.11
Docosapentaenoic (C22:5n-3)	0.03	0.04	0.04	0.10	0.13	0.14
Total PUFA	62.51	63.93	65.15	62.33	64.72	65.84
n-6/n-3 PUFA ratio	11.09	3.37	1.76	11.47	3.04	1.75
Linoleic (C18:2n-6)/α-linolenic (C18:3n-3) ratio	11.40	3.38	1.75	11.99	3.04	1.74

* The composition premix per kilogram: retinol—2,400,000 international units; calciferol—540,000 international units; α-tocopherol acetate—10,000 mg; phylloquinone—600 mg; thiamine—400 mg; riboflavin—1400 mg; pyridoxine—800 mg; cobalamin—4 mg; pantothenic acid—2000 mg; nicotinic acid—6000 mg; folic acid—200 mg; biotin—10 mg; calcium—121 g, iron—5000 mg; zinc—12,000 mg; manganese—2000 mg; copper—1000 mg; selenium—50 mg; iodine—250 mg; 6-phytase—200,000 phytase units; endo-1.4-beta-xylanase—480,000 units; citric acid—270 mg clinoptilolite—26.622 g, lysine—200 g; and threonine—210 g. C—diet with soybean meal (control); LIN6—diet with 6% linseed cake; LIN12—diet with 12% linseed cake; SFA—saturated fatty acids; MUFA—monounsaturated fatty acids; PUFA—polyunsaturated fatty acids; and dig.—digestible.

**Table 2:** Nutrient value and fatty acid composition of linseed cakes.

Item	Content
Dry matter, %	91.12
Metabolizable energy, MJ/kg	14.04
Crude protein, %	32.50
Lysine (dig.), g/kg	9.63
Methionine + Cystine (dig.), g/kg	9.94
Threonine (dig.), g/kg	10.93
Tryptophan (dig.), g/kg	4.33
Fat, %	17.51
Fiber, %	14.95
Ash, %	4.96
Calcium, %	0.18
Phosphorus, %	0.80
Fatty acids (% of total fatty acids)	
SFA	10.61
MUFA	18.86
n-6 PUFA	15.83
Linoleic (C18:2n-6)	15.36
n-3 PUFA	54.70
α-linolenic (C18:3n-3)	54.53
Eicosapentaenoic (C20:5n-3)	0.10
Docosapentaenoic (C22:5n-3)	0.07
Total PUFA	70.53
n-6/n-3 PUFA ratio	0.29
Linoleic (C18:2n-6)/α-linolenic (C18:3n-3) ratio	0.28

SFA—saturated fatty acid; MUFA—monounsaturated fatty acid; PUFA—polyunsaturated fatty acid; and dig.—digestible.

**Table 3:** Effect of dietary linseed cake on the growth performance of broiler chickens.

Item	Group			SED	*p*-Value
	C	LIN6	LIN12		
Live weight, g					
14 days	582.71	584.85	578.54	11.436	0.875
21 days	1067.05	1068.17	1026.51	20.009	0.084
28 days	1639.77	1604.73	1556.08	36.240	0.132
35 days	2254.34 ^a^	2185.02 ^ab^	2088.46 ^b^	50.863	0.014
42 days	3043.03 ^a^	2935.33 ^a^	2741.23 ^b^	75.814	<0.001
Feed intake (kg bird):					
15–28 day	1.596	1.576	1.508	0.040	0.218
29–42 day	2.618 ^a^	2.595 ^a^	2.268 ^b^	0.050	<0.001
15–42 day	4.214 ^a^	4.171 ^a^	3.776 ^b^	0.061	<0.001
Feed conversion ratio:					
15–28 day	1.507	1.545	1.542	0.025	0.254
29–42 day	1.892	1.963	1.923	0.088	0.723
15–42 day	1.717	1.779	1.748	0.027	0.244

C—diet with soybean meal (control); LIN6—diet with 6% linseed cake; LIN12—diet with 12% linseed cake; and SED—standard error of difference. ^a,b^—means in the same row with different superscripts differ significantly at *p* < 0.05.

**Table 4:** Effect of dietary linseed cake on chicken carcass dissection data.

Item	Group			SED	*p*-Value
	C	LIN6	LIN12		
Pre-slaughter weight, g	3378.67 ^a^	3293.50 ^ab^	3053.42 ^b^	105.226	0.008
Carcass weight, g	2541.56 ^a^	2437.71 ^a^	2246.52 ^b^	83.242	0.012
Carcass yield, %	75.24 ^a^	74.00 ^b^	73.54 ^b^	0.456	0.014
Non-edible internal, %	5.09 ^c^	5.57 ^b^	6.15 ^a^	0.192	<0.001
Heart, %	0.55	0.56	0.59	0.025	0.287
Gizzard, %	1.43	1.39	1.50	0.073	0.352
Liver, %	1.98	1.97	2.09	0.079	0.273
Abdominal fat, %	0.91	0.85	0.75	0.146	0.533
Breast muscle, %	29.53	29.10	28.55	0.749	0.427
Thigh muscle, %	9.98	10.14	10.18	0.358	0.842
Drumstick muscle, %	7.05	6.72	7.20	0.235	0.118

C—diet with soybean meal (control); LIN6—diet with 6% linseed cake; LIN12—diet with 12% linseed cake; and SED—standard error of difference. ^a,b,c^—means in the same row with different superscripts differ significantly at *p* < 0.05.

**Table 5:** Chemical composition of broiler chicken meat (%).

Item	Group			SED	*p*-Value
	C	LIN6	LIN12		
Breast					
Dry matter	24.01	24.18	24.34	0.177	0.181
Protein	20.96	21.18	21.45	0.228	0.108
Fat	1.56 ^a^	1.29 ^b^	1.09 ^b^	0.092	<0.001
Ash	1.09	1.08	1.08	0.017	0.983
Thigh					
Dry matter	24.80	24.73	24.07	0.480	0.251
Protein	18.72	18.81	18.79	0.233	0.923
Fat	4.51	4.36	4.28	0.239	0.629
Ash	1.06 ^a^	0.98 ^b^	1.04 ^a^	0.016	<0.001

C—diet with soybean meal (control); LIN6—diet with 6% linseed cake; LIN12—diet with 12% linseed cake; and SED—standard error of difference. ^a,b^—means in the same row with different superscripts differ significantly at *p* < 0.05.

**Table 6:** Saturated and monounsaturated fatty acids in the breast (% of total fatty acids).

Fatty Acid	Group									*p*-Value (FDR Adj.)
	C			LIN6			LIN12			
	Mean		SE	Mean		SE	Mean		SE	
Lauric (C12:0)	0.03	^c^	0.003	0.04	^b^	0.003	0.05	^a^	0.002	0.039
Myristic (C14:0)	0.37	^a^	0.004	0.21	^c^	0.004	0.30	^b^	0.007	0.039
Pentadecanoic (C15:0)	1.35	^b^	0.035	1.57	^a^	0.079	1.67	^a^	0.049	0.039
Palmitic (C16:0)	22.12	^a^	0.119	19.81	^b^	0.101	18.86	^c^	0.097	0.039
Margaric (C17:0)	0.07		0.011	0.11		0.005	0.10		0.010	0.330
Stearic (C18:0)	6.18	^b^	0.063	7.02	^a^	0.094	7.12	^a^	0.113	0.039
Arachidic (C20:0)	0.03	^b^	0.003	0.04	^b^	0.002	0.06	^a^	0.002	0.039
Behenic (C22:0)	0.09	^a^	0.005	0.08	^a^	0.006	0.06	^b^	0.003	0.039
Total SFA	30.24	^a^	0.113	28.99	^b^	0.165	28.21	^c^	0.208	0.039
Myristoleic (C14:1n-7)	0.09		0.003	0.06		0.004	0.09		0.008	0.080
Palmitelaidic (C16:1n-7t)	0.03	^b^	0.001	0.03	^b^	0.001	0.04	^a^	0.002	0.039
Hexadecenoic (C16:1n-9)	0.38		0.020	0.38		0.014	0.35		0.013	0.039
Palmitoleic (C16:1n-7)	3.79	^a^	0.077	2.26	^b^	0.057	1.86	^c^	0.031	0.039
Heptadecenoic (C17:1n-9)	0.18		0.010	0.18		0.008	0.20		0.009	0.039
Elaidic (C18:1n-9t)	0.11		0.006	0.10		0.003	0.10		0.002	1.000
Oleic (C18:1n-9)	33.28		0.150	30.03		0.189	27.07		0.126	1.000
Vaccenic (C18:1n-7)	2.20	^a^	0.020	1.92	^b^	0.023	1.81	^b^	0.035	0.039
Eicosenoic (C20:1n-9)	0.08	^b^	0.006	0.21	^a^	0.013	0.18	^a^	0.009	0.039
Erucic (C22:1n-9)	0.00	^b^	0.000	0.04	^a^	0.005	0.06	^a^	0.009	0.039
Total MUFA	40.15	^a^	0.148	35.21	^b^	0.205	31.76	^c^	0.156	0.039

C—diet with soybean meal (control); LIN6—diet with 6% linseed cake; LIN12—diet with 12% linseed cake; SFA—saturated fatty acids; and MUFA—monounsaturated fatty acids. Within a row, means with different superscript letters differ significantly (*p* < 0.05). *p*-values were adjusted for multiple testing, using the Benjamini–Hochberg false discovery rate (FDR) procedure.

**Table 7:** Saturated and monounsaturated fatty acids in the thigh (% of total fatty acids).

Fatty Acid	Group									*p*-Value (FDR Adj.)
	C			LIN6			LIN12			
	Mean		SE	Mean		SE	Mean		SE	
Lauric (C12:0)	0.02		0.002	0.02		0.002	0.02		0.003	1.000
Myristic (C14:0)	0.38	^a^	0.004	0.34	^b^	0.005	0.31	^c^	0.005	0.040
Pentadecanoic (C15:0)	0.54		0.029	0.61		0.031	0.52		0.028	1.000
Palmitic (C16:0)	21.38	^a^	0.153	18.68	^b^	0.090	17.31	^c^	0.091	0.040
Margaric (C17:0)	0.08		0.004	0.06		0.003	0.06		0.004	0.110
Stearic (C18:0)	5.58		0.098	5.60		0.044	5.60		0.070	1.000
Arachidic (C20:0)	0.03		0.003	0.03		0.001	0.04		0.004	1.000
Behenic (C22:0)	0.09	^a^	0.005	0.07	^a^	0.004	0.05	^b^	0.003	0.040
Total SFA	28.10	^a^	0.165	25.41	^b^	0.109	23.91	^c^	0.088	0.040
Myristoleic (C14:1n-7)	0.12	^a^	0.007	0.08	^b^	0.003	0.06	^c^	0.004	0.040
Palmitelaidic (C16:1n-7t)	0.02	^b^	0.001	0.03	^a^	0.001	0.03	^a^	0.001	0.040
Hexadecenoic (C16:1n-9)	0.37		0.021	0.34		0.012	0.27		0.014	0.100
Palmitoleic (C16:1n-7)	4.57	^a^	0.088	3.55	^b^	0.071	2.93	^c^	0.077	0.040
Heptadecenoic (C17:1n-9)	0.09		0.004	0.10		0.002	0.09		0.006	0.310
Elaidic (C18:1n-9t)	0.11	^a^	0.008	0.09	^a^	0.003	0.05	^b^	0.002	0.040
Oleic (C18:1n-9)	34.71	^a^	0.195	32.69	^b^	0.335	32.06	^b^	0.171	0.040
Vaccenic (C18:1n-7)	2.00	^b^	0.020	2.39	^a^	0.074	1.78	^c^	0.018	0.040
Eicosenoic (C20:1n-9)	0.26	^a^	0.007	0.20	^b^	0.013	0.18	^b^	0.010	0.040
Erucic (C22:1n-9)	0.03	^a^	0.005	0.02	^a^	0.002	0.01	^b^	0.001	0.040
Total MUFA	42.28	^a^	0.224	39.48	^b^	0.316	37.46	^c^	0.194	0.040

C—diet with soybean meal (control); LIN6—diet with 6% linseed cake; LIN12—diet with 12% linseed cake; SFA—saturated fatty acids; and MUFA—monounsaturated fatty acids. Within a row, means with different superscript letters differ significantly (*p* < 0.05). *p*-values were adjusted for multiple testing, using the Benjamini–Hochberg false discovery rate (FDR) procedure.

**Table 8:** Saturated and monounsaturated fatty acids in the abdominal fat (% of total fatty acids).

Fatty Acid	Group									*p*-Value (FDR Adj.)
	C			LIN6			LIN12			
	Mean		SE	Mean		SE	Mean		SE	
Lauric (C12:0)	0.02		0.001	0.03		0.001	0.02		0.001	0.540
Myristic (C14:0)	0.33	^a^	0.015	0.31	^b^	0.004	0.29	^b^	0.004	0.040
Pentadecanoic (C15:0)	0.08	^a^	0.003	0.07	^b^	0.002	0.06	^b^	0.004	0.040
Palmitic (C16:0)	21.64	^a^	0.192	19.04	^b^	0.110	17.30	^c^	0.132	0.040
Margaric (C17:0)	0.06	^a^	0.003	0.05	^a^	0.003	0.04	^b^	0.003	0.040
Stearic (C18:0)	4.78	^b^	0.060	5.25	^a^	0.083	4.88	^b^	0.104	0.180
Arachidic (C20:0)	0.04		0.001	0.06		0.009	0.06		0.008	1.000
Behenic (C22:0)	0.05	^a^	0.002	0.04	^a^	0.001	0.03	^b^	0.001	0.040
Total SFA	27.00	^a^	0.200	24.85	^b^	0.140	22.68	^c^	0.187	0.040
Myristoleic (C14:1n-7)	0.10	^a^	0.004	0.07	^b^	0.005	0.07	^b^	0.004	0.040
Palmitelaidic (C16:1n-7t)	0.03	^a^	0.001	0.03	^a. b^	0.003	0.02	^b^	0.001	0.040
Hexadecenoic (C16:1n-9)	0.21		0.018	0.20		0.011	0.18		0.009	1.000
Palmitoleic (C16:1n-7)	4.98	^a^	0.075	3.65	^b^	0.069	3.39	^b^	0.096	0.040
Heptadecenoic (C17:1n-9)	0.06		0.002	0.06		0.004	0.05		0.003	0.170
Elaidic (C18:1n-9t)	0.08	^a^	0.005	0.05	^b^	0.001	0.04	^c^	0.002	0.040
Oleic (C18:1n-9)	34.75	^a^	0.464	31.55	^b^	0.166	30.97	^b^	0.247	0.040
Vaccenic (C18:1n-7)	5.02		0.404	5.55		0.136	4.84		0.087	0.410
Eicosenoic (C20:1n-9)	0.21	^a^	0.005	0.18	^b^	0.003	0.19	^b^	0.016	0.040
Erucic (C22:1n-9)	0.01	^b^	0.001	0.02	^a^	0.001	0.02	^a^	0.001	0.040
Total MUFA	45.45	^a^	0.137	41.36	^b^	0.219	39.77	^c^	0.247	0.040

C—diet with soybean meal (control); LIN6—diet with 6% linseed cake; LIN12—diet with 12% linseed cake; SFA—saturated fatty acids; MUFA—monounsaturated fatty acids. Within a row, means with different superscript letters differ significantly (*p* < 0.05). *p*-values were adjusted for multiple testing using the Benjamini–Hochberg false discovery rate (FDR) procedure.

**Table 9:** Polyunsaturated fatty acids in the breast (% of total fatty acids).

Fatty Acid	Group									*p*-Value (FDR Adj.)
	C			LIN6			LIN12			
	Mean		SE	Mean		SE	Mean		SE	
Linolelaidic (C18:2n-6t)	0.05	^a^	0.001	0.04	^b^	0.001	0.04	^b^	0.003	0.039
Octadecadienoic (C18:2n-6c.t)	0.03		0.001	0.03		0.001	0.03		0.001	0.540
Octadecadienoic (C18:2n-6t.c)	0.02	^a^	0.001	0.02	^b^	0.001	0.01	^c^	0.001	0.039
Linoleic (C18:2n-6)	22.95	^b^	0.161	24.13	^a^	0.162	24.54	^a^	0.230	0.039
γ-linolenic (C18:3n-6)	0.20	^a^	0.007	0.17	^b^	0.006	0.15	^b^	0.004	0.039
Eicosadienoic (C20:2n-6)	0.33	^b^	0.013	0.43	^a^	0.022	0.39		0.009	0.039
Eicosatrienoic (C20:3n-6)	0.36		0.011	0.39		0.022	0.38		0.012	1.000
Arachidonic (C20:4n-6)	2.04	^b^	0.050	2.33	^a^	0.053	2.54	^a^	0.077	0.039
Docosatetraenoic (C22:4n-6)	0.62	^b^	0.017	0.69	^b^	0.013	0.78	^a^	0.021	0.039
Total n-6 PUFA	26.60	^b^	0.145	28.21	^a^	0.165	28.86	^a^	0.167	0.039
Total LC n-6 PUFA	3.35	^b^	0.083	3.83	^a^	0.082	4.09	^a^	0.108	0.039
α-linolenic (C18:3n-3)	1.55	^c^	0.025	4.97	^b^	0.101	7.74	^a^	0.097	0.039
Eicosatrienoic (C20:3n-3)	0.09	^b^	0.003	0.20	^a^	0.017	0.19	^a^	0.006	0.039
Eicosapentaenoic (C20:5n-3)	0.04		0.005	0.03	^b^	0.002	0.05	^a^	0.005	0.039
Docosapentaenoic (C22:5n-3)	0.32	^c^	0.008	0.82	^b^	0.038	1.31	^a^	0.028	0.039
Docosahexaenoic (C22:6n-3)	0.18	^c^	0.017	0.56	^b^	0.052	0.83	^a^	0.039	0.039
Total n-3 PUFA	2.19	^c^	0.029	6.59	^b^	0.065	10.13	^a^	0.077	0.039
Total LC n-3 PUFA	0.63	^c^	0.017	1.61	^b^	0.100	2.39	^a^	0.060	0.039
Total PUFA	28.79	^c^	0.157	34.80	^b^	0.190	38.99	^a^	0.189	0.039
Total trans acid	0.24	^a^	0.007	0.22	^b^	0.005	0.22		0.007	0.039
PUFA/SFA	0.95	^c^	0.008	1.20	^b^	0.012	1.38	^a^	0.016	0.039
PUFA/MUFA	0.72	^c^	0.006	0.99	^b^	0.010	1.23	^a^	0.010	0.039
n-6/n-3 PUFA ratio	12.20	^a^	0.156	4.29	^b^	0.045	2.85	^c^	0.027	0.039
Linoleic (C18:2n-6)/α-linolenic (C18:3n-3) ratio	14.84	^a^	0.244	4.89	^b^	0.093	3.18	^c^	0.030	0.039

C—diet with soybean meal (control); LIN6—diet with 6% linseed cake; LIN12—diet with 12% linseed cake; SFA—saturated fatty acids; MUFA—monounsaturated fatty acids; PUFA—polyunsaturated fatty acids; and LC—long chain. Within a row, means with different superscript letters differ significantly (*p* < 0.05). *p*-values were adjusted for multiple testing, using the Benjamini–Hochberg false discovery rate (FDR) procedure.

**Table 10:** Polyunsaturated fatty acids in the thigh muscle (% of total fatty acids).

Fatty Acid	Group									*p*-Value (FDR Adj.)
	C			LIN6			LIN12			
	Mean		SE	Mean		SE	Mean		SE	
Linolelaidic (C18:2n-6t)	0.05	^a^	0.002	0.03	^b^	0.001	0.04	^b^	0.001	0.040
Octadecadienoic (C18:2n-6c.t)	0.03		0.001	0.03		0.001	0.03		0.001	0.644
Octadecadienoic (C18:2n-6t.c)	0.02	^a^	0.001	0.02	^a^	0.001	0.01	^b^	0.001	0.040
Linoleic (C18:2n-6)	24.39	^b^	0.233	25.55		0.261	25.97	^a^	0.157	0.040
γ-linolenic (C18:3n-6)	0.23	^a^	0.004	0.17	^b^	0.004	0.18	^b^	0.004	0.040
Eicosadienoic (C20:2n-6)	0.21		0.007	0.24		0.006	0.25		0.009	0.100
Eicosatrienoic (C20:3n-6)	0.21		0.008	0.21		0.006	0.20		0.009	1.000
Arachidonic (C20:4n-6)	1.42		0.059	1.59		0.053	1.37		0.027	0.100
Docosatetraenoic (C22:4n-6)	0.45		0.027	0.45		0.020	0.45		0.016	1.000
Total n-6 PUFA	27.02	^b^	0.257	28.28	^a^	0.264	28.49	^a^	0.175	0.040
Total LC n-6 PUFA	2.30	^b^	0.093	2.48	^a^	0.061	2.27	^b^	0.041	0.040
α-linolenic (C18:3n-3)	1.61	^c^	0.025	5.48	^b^	0.067	8.74	^a^	0.083	0.040
Eicosatrienoic (C20:3n-3)	0.03	^b^	0.002	0.10	^a^	0.004	0.11	^a^	0.004	0.040
Eicosapentaenoic (C20:5n-3)	0.04		0.002	0.02		0.001	0.03		0.002	0.100
Docosapentaenoic (C22:5n-3)	0.23	^b^	0.014	0.48	^a^	0.018	0.52	^a^	0.016	0.040
Docosahexaenoic (C22:6n-3)	0.15	^c^	0.016	0.25	^b^	0.010	0.31	^a^	0.010	0.040
Total n-3 PUFA	2.05	^c^	0.032	6.32	^b^	0.088	9.70	^a^	0.096	0.040
Total LC n-3 PUFA	0.44	^c^	0.032	0.84	^b^	0.029	0.97	^a^	0.025	0.040
Total PUFA	29.07	^c^	0.273	34.60	^b^	0.278	38.19	^a^	0.184	0.040
Total trans acid	0.23	^a^	0.011	0.20	^b^	0.005	0.16	^c^	0.004	0.040
PUFA/SFA	1.04	^c^	0.014	1.36	^b^	0.012	1.60	^a^	0.011	0.040
PUFA/MUFA	0.69	^c^	0.009	0.88	^b^	0.014	1.02	^a^	0.010	0.040
n-6/n-3 PUFA ratio	13.24	^a^	0.189	4.50	^b^	0.078	2.94	^c^	0.036	0.040
Linoleic (C18:2n-6)/α-linolenic (C18:3n-3) ratio	15.27	^a^	0.242	4.69	^b^	0.083	2.98	^c^	0.038	0.040

C—diet with soybean meal (control); LIN6—diet with 6% linseed cake; LIN12—diet with 12% linseed cake; SFA—saturated fatty acids; MUFA—monounsaturated fatty acids; PUFA—polyunsaturated fatty acids; and LC—long chain. Within a row, means with different superscript letters differ significantly (*p* < 0.05). *p*-values were adjusted for multiple testing, using the Benjamini–Hochberg false discovery rate (FDR) procedure.

**Table 11:** Polyunsaturated fatty acids in the abdominal fat (% of total fatty acids).

Fatty Acid	Group									*p*-Value (FDR Adj.)
	C			LIN6			LIN12			
	Mean		SE	Mean		SE	Mean		SE	
Linolelaidic (C18:2n-6t)	0.02		0.001	0.03		0.001	0.03		0.001	0.050
Octadecadienoic (C18:2n-6c.t)	0.02		0.001	0.02		0.001	0.02		0.001	0.280
Octadecadienoic(C18:2n-6t.c)	0.02	^a^	0.002	0.01	^b^	0.000	0.01	^b^	0.000	0.040
Linoleic (C18:2n-6)	24.69	^c^	0.165	26.42	^b^	0.162	26.64	^a^	0.226	0.040
γ-linolenic (C18:3n-6)	0.23	^a^	0.003	0.18	^b^	0.005	0.16		0.005	0.040
Eicosadienoic (C20:2n-6)	0.13	^b^	0.003	0.17	^a^	0.003	0.16		0.008	0.040
Eicosatrienoic (C20:3n-6)	0.09		0.003	0.09	^a^	0.002	0.08	^b^	0.002	0.040
Arachidonic (C20:4n-6)	0.21	^b^	0.004	0.23	^a^	0.009	0.22		0.008	0.040
Docosatetraenoic (C22:4n-6)	0.05	^b^	0.002	0.08	^a^	0.005	0.13	^a^	0.010	0.040
Total n-6 PUFA	25.46	^b^	0.165	27.22	^a^	0.166	27.44	^a^	0.244	0.040
Total LC n-6 PUFA	0.48	^b^	0.006	0.57	^a^	0.012	0.58	^a^	0.021	0.040
α-linolenic (C18:3n-3)	1.64	^c^	0.023	6.01	^b^	0.070	9.43	^a^	0.099	0.040
Eicosatrienoic (C20:3n-3)	0.03		0.002	0.03		0.001	0.03		0.003	0.990
Eicosapentaenoic (C20:5n-3)	0.04	^b^	0.001	0.10	^a^	0.006	0.13	^a^	0.016	0.040
Docosapentaenoic (C22:5n-3)	0.04	^c^	0.003	0.06	^b^	0.004	0.12	^a^	0.005	0.040
Docosahexaenoic (C22:6n-3)	0.07		0.003	0.08		0.004	0.08		0.003	1.000
Total n-3 PUFA	1.82	^c^	0.023	6.28	^b^	0.074	9.80	^a^	0.103	0.040
Total LC n-3 PUFA	0.18	^c^	0.005	0.26	^b^	0.009	0.37	^a^	0.019	0.040
Total PUFA	27.28		0.166	33.50		0.189	37.24		0.258	1.000
Total trans acid	0.17	^a^	0.007	0.14	^b^	0.003	0.12	^c^	0.002	0.040
PUFA/SFA	1.01	^c^	0.013	1.35	^b^	0.012	1.65	^a^	0.021	0.040
PUFA/MUFA	0.60	^c^	0.004	0.81	^b^	0.008	0.94	^a^	0.011	0.040
n-6/n-3 PUFA ratio	14.05	^a^	0.203	4.35	^b^	0.056	2.81	^c^	0.042	0.040
Linoleic (C18:2n-6)/α-linolenic (C18:3n-3) ratio	15.10	^a^	0.229	4.41	^b^	0.056	2.83	^c^	0.041	0.040

C—diet with soybean meal (control); LIN6—diet with 6% linseed cake; LIN12—diet with 12% linseed cake; SFA—saturated fatty acids; MUFA—monounsaturated fatty acids; PUFA—polyunsaturated fatty acids; and LC—long chain. Within a row, means with different superscript letters differ significantly (*p* < 0.05). *p*-values were adjusted for multiple testing, using the Benjamini–Hochberg false discovery rate (FDR) procedure.

**Table 12:** Indices in the breast, thigh, and abdominal fat (% of total fatty acids).

Item	Group									*p*-Value (FDR Adj.)
	C			LIN6			LIN12			
	Mean		SE	Mean		SE	Mean		SE	
Breast										
Atherogenic index	0.34	^a^	0.002	0.30	^b^	0.002	0.28	^c^	0.002	0.039
Trombogenic index	0.72	^a^	0.005	0.53	^b^	0.004	0.43	^c^	0.004	0.039
Hypo/hypercholesterolemic index	2.72	^c^	0.017	3.17	^b^	0.020	3.40	^a^	0.026	0.039
Hipercholesterolemic saturated fatty acids, %	23.63	^a^	0.114	21.16	^b^	0.105	20.10	^c^	0.098	0.039
Desirable fatty acids, %	75.12	^c^	0.093	77.03	^b^	0.122	77.87	^a^	0.128	0.039
Peroxidability index	42.07	^c^	0.282	57.74	^b^	0.634	70.05	^a^	0.498	0.039
Unsaturation index	107.07	^c^	0.253	121.35	^b^	0.450	132.26	^a^	0.280	0.039
Thigh										
Atherogenic index	0.32	^a^	0.003	0.27	^b^	0.002	0.25	^c^	0.001	0.040
Trombogenic index	0.67	^a^	0.006	0.46	^b^	0.003	0.37	^c^	0.002	0.040
Hypo/hypercholesterolemic index	2.90	^c^	0.026	3.50	^b^	0.020	3.95	^a^	0.026	0.040
Hipercholesterolemic saturated fatty acids, %	22.92	^a^	0.160	20.05	^b^	0.095	18.59	^c^	0.096	0.040
Desirable fatty acids, %	76.93	^c^	0.137	79.68	^b^	0.095	81.25	^a^	0.081	0.040
Peroxidability index	40.18	^c^	0.553	51.87	^b^	0.505	58.69	^a^	0.376	0.040
Unsaturation index	107.63	^c^	0.516	121.19	^b^	0.415	129.57	^a^	0.341	0.040
Abdominal fat										
Atherogenic index	0.32	^a^	0.003	0.27	^b^	0.002	0.24	^c^	0.002	0.040
Trombogenic index	0.55	^a^	0.002	0.39	^b^	0.003	0.31	^c^	0.002	0.040
Hypo/hypercholesterolemic index	2.81	^c^	0.049	3.34	^b^	0.025	3.86	^a^	0.039	0.040
Hipercholesterolemic saturated fatty acids, %	22.98	^a^	0.182	20.32	^b^	0.120	18.50	^c^	0.141	0.040
Desirable fatty acids, %	77.51	^c^	0.185	80.11	^b^	0.116	81.89	^a^	0.140	0.040
Peroxidability index	32.08	^c^	0.172	43.11	^b^	0.249	50.82	^a^	0.330	0.040
Unsaturation index	103.05	^c^	0.340	116.08	^b^	0.306	125.74	^a^	0.464	0.040

C—diet with soybean meal (control); LIN6—diet with 6% linseed cake; LIN12—diet with 12% linseed cake; and SE—standard error. Within a row, means with different superscript letters differ significantly (*p* < 0.05). *p*-values were adjusted for multiple testing, using the Benjamini–Hochberg false discovery rate (FDR) procedure.

## Data Availability

Data are contained within the article.

## Abbreviations

The following abbreviations are used in this manuscript:

C	The control group, diet with soybean meal
LIN 6	Diet with 6% linseed cake
LIN 12	Diet with 12% linseed cake
PUFA	Polyunsaturated Fatty Acids
ALA	Alpha-linolenic acid
LC n-3 PUFA	Long chain n-3 PUFA
EPA	Eicosapentaenoic fatty acid (C20:5n-3)
DHA	Docosahexaenoic fatty acid (C22:6n-3)
SFA	Saturated fatty acids
MUFA	Monounsaturated fatty acids
AOAC	Association of Official Analytical Chemists
MJ	Metabolizable energy
HPLC	High-performance liquid chromatography
FDR	False discovery rate
FAME	Fatty acid methyl esters
AI	Atherogenic index
TI	Thrombogenic index
h/H	hypocholesterolemic/Hypercholesterolemic
PI	Peroxidability index
UI	Unsaturation index
DFA	Desirable fatty acids
HSFA	Hypercholesterolemic saturated fatty acids
DM	Dry matter
OM	Organic matter
CP	Crude protein
CF	Crude fat
GLM	General linear model
FCR	Feed conversion ratio
NFE	Nitrogen-free extract
LDL	Low-density lipoprotein
